# Identification of a functional variant for colorectal cancer risk mapping to chromosome 5q31.1

**DOI:** 10.18632/oncotarget.9298

**Published:** 2016-05-11

**Authors:** Juntao Ke, Jiao Lou, Xueqin Chen, Jiaoyuan Li, Cheng Liu, Yajie Gong, Yang Yang, Ying Zhu, Yi Zhang, Jianbo Tian, Jiang Chang, Rong Zhong, Jing Gong, Xiaoping Miao

**Affiliations:** ^1^ Department of Epidemiology and Biostatistics, School of Public Health, Tongji Medical College, Huazhong University of Science and Technology, Wuhan, China; ^2^ State Key Laboratory of Environment Health (Incubation), MOE (Ministry of Education) Key Laboratory of Environment and Health, Ministry of Environmental Protection Key Laboratory of Environment and Health (Wuhan) and School of Public Health, Tongji Medical College, Huazhong University of Science and Technology, Wuhan, China

**Keywords:** a functional variant, rs17716310, colorectal cancer, chromosome 5q31.1

## Abstract

Genome-wide association studies (GWASs) have established chromosome 5q31.1 as a risk locus for colorectal cancer (CRC). We previously identified a potentially regulatory single nucleotide polymorphism (SNP) rs17716310 within 5q31.1. Now, we extended our study with another independent Chinese population, functional assays and analyses of TCGA (The Cancer Genome Atlas) data. Significant associations between rs17716310 and CRC risk were found in Present Study including 1075 CRC cases and 1999 controls (additive model: OR = 1.149, 95% CI = 1.027–1.286, *P* = 0.016), and in Combined Study including 1766 cases and 2708 controls (additive model: OR = 1.145, 95% CI = 1.045–1.254, *P* = 0.004). Dual luciferase reporter gene assays indicated that the variant C allele obviously increased transcriptional activity. Using TCGA datasets, we indicated rs17716310 as a cis expression quantitative trait locus (eQTL) for the gene *SMAD5*, whose expression was significantly higher in CRC tissues. These findings suggested that the functional polymorphism rs17716310 A > C might be a genetic modifier for CRC, promoting the expression of *SMAD5* that belonged to the transforming growth factor beta (TGF-β) signaling pathway.

## INTRODUCTION

Colorectal cancer (CRC) is the third most commonly diagnosed cancer in males and the second in females in the worldwide [1]. In China, the incidence and mortality of CRC have rapidly increased in the past ten years, with an estimated 310,244 new cases and 149,722 deaths occurring in 2011 [2, 3]. In addition to environment factors like diet, obesity, physical inactivity, cigarette smoking and alcohol consumption [4, 5], genetics has been well established as an important factor in CRC etiology [6–8]. Large-scale genome-wide association studies (GWASs) and following researches have identified numerous CRC-associated single nucleotide polymorphisms (SNPs) in over 30 chromosome loci [9–23]. However, most risk variants are located in non-coding regions without clear biological mechanisms [24], and the functional and causal SNPs remain to be mined.

At the same time, it has been proved that the identification of functional SNPs could be facilitated with the application of regulatory elements predicted by chromatin status like histone modifications [25–28]. For example, using their own chromatin immunoprecipitation-sequencing (ChIP-seq) data of histone modifications and other data from Encyclopedia of DNA Elements (ENCODE), Biancolella *et al.* identified rs10891246 and rs7130173 as functional SNPs mapping to CRC GWAS locus 11q23.1. They also indicated *C11orf53*, *C11orf92* and *C11orf93* as novel candidate genes for CRC risk [29].

Chromosome 5q31.1 was discovered and replicated as a CRC susceptibility locus by Jia *et al.* [20] and Zhang *et al.* [23], and the reported strongest risk SNPs rs647161 is of unclear function. To refine this region, we searched potentially functional SNPs in regulatory elements indicated by CRC-specific histone modifications. And we found rs17716310 confer significantly and marginally increase risk for CRC in a Chinese population [30]. Here we continued the previous study with another independent Chinese population including 1075 cases and 1999 controls, and a Combined Study including 1766 cases and 2708 controls. Furthermore, we verified the functionality of rs17716310 by the dual luciferase reporter gene assays and the analyses of TCGA (The Cancer Genome Atlas) data.

## RESULTS

### Population characteristics

Characteristics of the study subjects were summarized in the Table 1. 1075 incident cases and 1999 controls were enrolled in the present validation study. Combing the samples of the discovery stage [30], 1766 cases and 2708 controls were included in the combined study. In both stages, cases and controls are adequately matched in terms of gender and age (*P* > 0.05). Significantly more smokers were found among the cases than controls in either stage (Present Study: 40.6% versus 34.5%, *P* = 0.001; Combined Study: 38.4% versus 33.2%, *P* = 4.308E-04). According to the calculations adjusted by sex and age, smokers in our study owned a higher risk for CRC than non-smokers (Present Study: OR = 1.385, 95% CI = 1.154–1.661; Combined Study: OR = 1.332, 95% CI = 1.147–1.546).

**Table 1:** The characteristics of the study population

	Present Study				Combined Study			
	Case (%)	Control (%)	χ^2^	*P*	Case (%)	Control (%)	χ^2^	*P*
Total	1075	1999			1766	2708		
Gender			0.707	0.400^a^			0.892	0.345^a^
Male	646 (60.1)	1170 (58.5)			1049 (59.4)	1570 (58.0)		
Female	429 (39.9)	829 (41.5)			717 (40.6)	1138 (42.0)		
Age (mean ± SD)	60.51 ± 12.82	61.07 ± 11.97		0.232^b^	60.36 ± 12.62	60.79 ± 12.30		0.254^b^
Agegroup			3.959	0.266^a^			0.718	0.869^a^
≤ 50	237 (22.1)	460 (23.0)			386 (21.9)	614 (22.7)		
51–60	281 (26.1)	574 (28.7)			487 (27.6)	755 (27.9)		
61–70	293 (27.3)	494 (24.7)			475 (26.9)	703 (26.0)		
≥ 71	263 (24.5)	471 (23.6)			417 (23.6)	636 (23.5)		
Smoking Status			10.990	**0.001^a^**			12.723	**4.308E–04**^a^
Non-Smoker	639 (59.4)	1309 (65.5)			1087 (61.6)	1808 (66.8)		
Smoker	436 (40.6)	690 (34.5)			679 (38.4)	900 (33.2)		

Abbreviations: SD, standard deviation.

a *P* value was calculated by chi-square test.

b *P* value was calculated by the *t* test.

The nominal significant results were in bold.

### Association analysis

Shown in Table 2, rs17716310 was evidently associated with CRC risk in the present validation study and in the combined study.

**Table 2:** Association between individual SNP and colorectal cancer risk

	Present Study				Combined Study			
	Cases (%)	Controls (%)	OR (95% CI)^a^	*P*^a^	Cases (%)	Control (%)^s^	OR (95% CI)^a^	*P*^a^
**rs17716310**								
AA	481 (45.2)	951 (48.1)	1.000		775 (44.4)	1289 (48.2)	1.000	
AC	454 (42.7)	842 (42.6)	1.071 (0.914–1.256)	0.397	768 (44.0)	1126 (42.1)	**1.144 (1.006–1.300)**	**0.041**
CC	129 (12.1)	183 (9.3)	**1.420 (1.104–1.827)**	**0.006**	203 (11.6)	259 (9.7)	**1.313 (1.070–1.612)**	**0.009**
Dominant			1.133 (0.975–1.316)	0.104			**1.175 (1.041–1.327)**	**0.009**
Recessive			**1.374 (1.081–1.747)**	**0.009**			**1.231 (1.012–1.496)**	**0.037**
Additive			**1.149 (1.027–1.286)**	**0.016**			**1.145 (1.045–1.254)**	**0.004**

Abbreviations: OR, Odds ratio; 95% CI, 95% confidence interval.

a Data were calculated by logistic regression model after adjusting for sex, age group and smoking status.

The nominal significant results were in bold.

Under multivariable logistic regression model adjusted for gender, age group and smoking status, individuals with CC genotype of rs17716310 had a significantly increased risk of CRC compared to those with AA homozygote (homozygous model: OR = 1.420, 95% CI = 1.104–1.827, *P* = 0.006), and to those with AA and AC (recessive model: OR = 1.374, 95% CI = 1.081–1.747, *P* = 0.009). Likewise, positive outcome was found in the additive models, with per-A-allele OR of 1.149 (95% CI = 1.027–1.286, *P* = 0.016).

To expand sample size and improve statistical power, we combined our previously published data [30] with aforementioned data into a pooled analysis (Combined Study). Significant associations were exhibited between rs17716310 and CRC risk in all genetic models we investigated (Heterozygous model: OR = 1.144, 95% CI = 1.006–1.300, *P*= 0.041; Homozygous model: OR = 1.313, 95% CI = 1.070–1.613, *P* = 0.009; Dominant model: OR = 1.175, 95% CI = 1.041–1.327, *P* = 0.009; Recessive model: OR = 1.231, 95% CI = 1.012–1.496, *P* = 0.037; Additive model: OR = 1.145, 95% CI = 1.046–1.254, *P* = 0.004). The results of stratified analyses by gender and median age were presented in Table S1.

Table 3 detailed the results of interaction analysis between rs17716310 and smoke in Combined Study, where we observed a significant interaction in multiplicative terms (*P*_mult_ = 0.003), but not in additive terms (*P*_add_ = 0.509). Individuals carrying rs17716310 variant genotypes showed an association with the risk of CRC, especially for smokers.

**Table 3:** Interaction analysis between smoking and rs17716310 associated with CRC risk in combined study

Smoking Status	Genotype	Case/Control	OR (95% CI)^a^	*P*_mult_^a^	*P*_add_
Non-smoker	AA	470/856	1.000	**0.003**	0.509
	AC + CC	601/925	1.186 (1.019–1.382)		
Smoker	AA	304/432	1.363 (1.114–1.669)		
	AC + CC	369/460	1.560 (1.281–1.898)		

*P*_mult_ was calculated using the multiplicative interaction term.

*P*_add_ was calculated using the additive interaction model.

a Data were calculated by logistic regression model after adjusting for gender and age group.

The nominal significant results were in bold.

### Dual luciferase reporter gene assay

**Figure 1:**
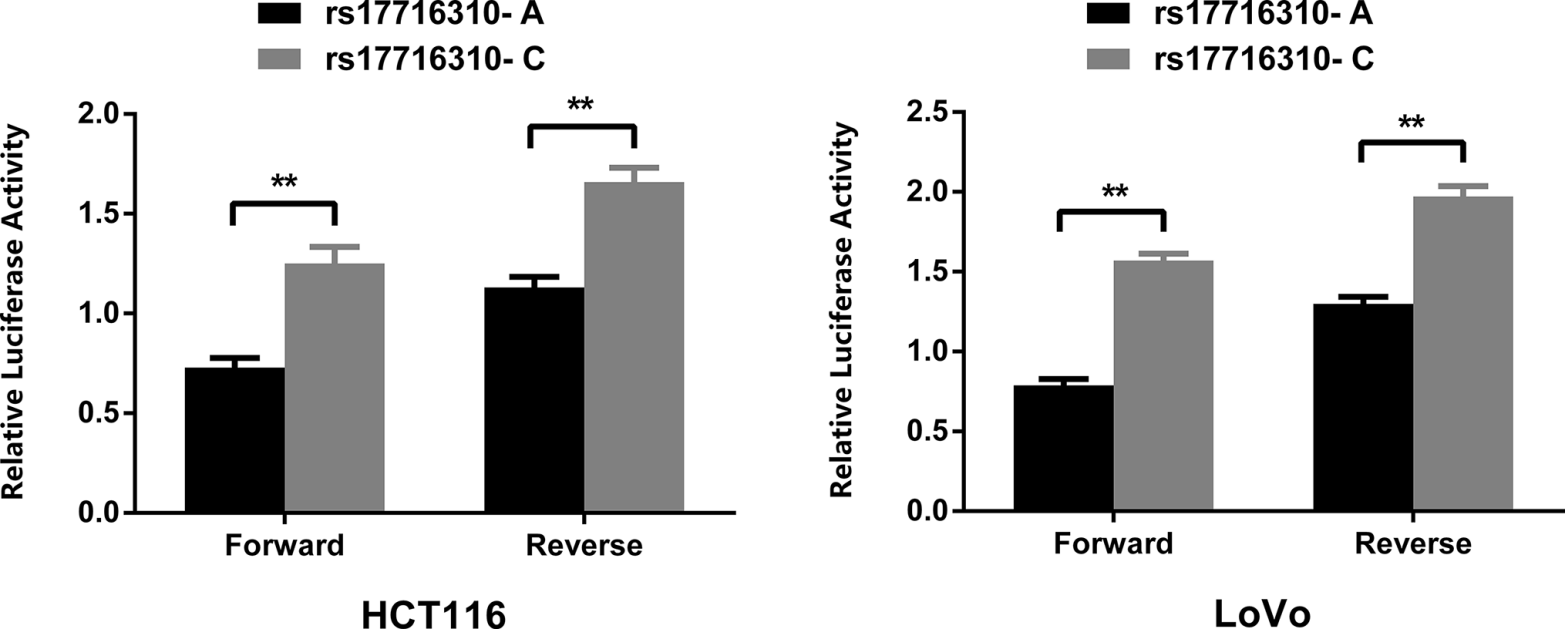
Reporter gene assays with two constructs containing major and minor alleles of rs17716310 in HCT116 and LoVo Both constructs were cotransfected with pRL-SV40 to standardize transfection efficiency. Luciferase levels of pGL3-promoter and pRL-SV40 were determined in triplicate. Data shown are the from three independent transfection experiments, each performed in triplicate. The rs17716310 C-containing enhancer drove significantly higher reporter gene expression than the rs17716310 A-containing fragment in both CRC cell lines (**P < 0.01).

We generated two luciferase reporter plasmids containing rs17716310 A and C allele, respectively, and used pRL-SV40 plasmids as normalized controls. In either CRC cell line HCT116 or LoVo, luciferase expression was significantly higher in the mutant C allelic construct compared with the major A construct in both forward and reverse sequence directions (*P* < 0.01, Figure 1). It showed that rs17716310 A > C could upregulate gene expression by increasing the transcriptional activity.

### eQTL analyses

**Figure 2:**
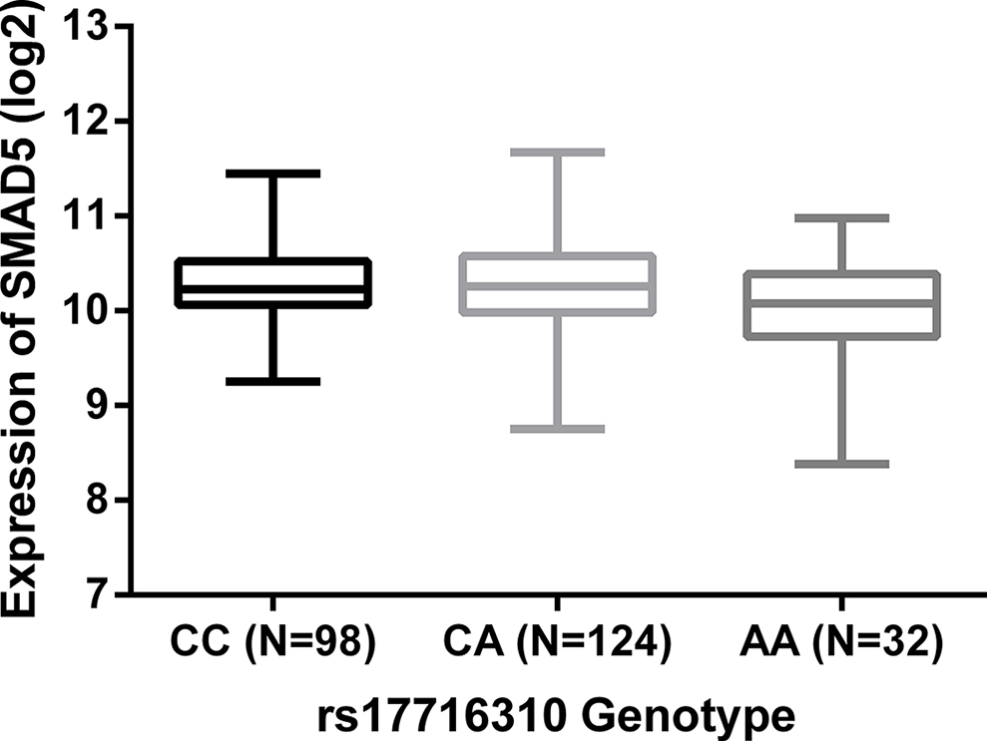
Expression correlation between rs17716310 and *SMAD5* The C allele of rs17716310 was correlated with higher SMAD5 expression under an adjusted linear regression model (*P* < 0.05). The values (average ± SD) of expression level (log^2^) were 10.29 ± 0.42 for CC, 10.28 ± 0.46 for CA and 10.06 ± 0.54 for AA.

Applying the multi-level TCGA datasets for COAD and READ, we performed an adjusted eQTL-analysis of the association between rs17716310 and expression of 23 genes in flanking 1Mb region. Shown in Table 4 and Figure 2, rs17716310 was identified as a cis-eQTL for the gene *SMAD5* (mothers against decapentaplegic homolog 5, *P* < 0.05), and its C allele was correlated with higher *SMAD5* expression. In addition, we compared *SMAD5* expression between 347 cancer and 50 adjacent normal tissues, and found significantly greater expression in CRC samples (*P* = 6.56 × 10^−7^, CRC tissue: 1323 ± 423 RPKM (reads per kilobases per million reads), peritumor tissue: 1020 ± 294 RPKM). When the expression of *SMAD5* was compared between 32 CRC tissues and paired peritumor tissues, obviously higher expression was still observed in cancerous tissues (*P* = 0.002, CRC tissue: 1421 ± 376 RPKM, paired peritumor tissue: 1109 ± 312 RPKM).

**Table 4:** Expression correlation between rs17716310 and flanking 1 Mb genes

Gene	Correlation *P*	Correlation R^2^
***SMAD5***	**4.997E–02**	**1.516E–02**
*SAR1B*	1.311E–01	9.020E–03
*UBE2B*	1.371E–01	8.749E–03
*DDX46*	1.426E–01	8.510E–03
*C5orf24*	1.694E–01	7.478E–03
*CAMLG*	2.307E–01	5.695E–03
*LECT2*	3.019E–01	4.229E–03
*PCBD2*	3.151E–01	4.004E–03
*CATSPER3*	3.281E–01	3.796E–03
*TXNDC15*	4.023E–01	2.786E–03
*TGFBI*	4.380E–01	2.388E–03
*PPP2CA*	4.524E–01	2.242E–03
*SEC24A*	5.204E–01	1.641E–03
*PHF15*	5.212E–01	1.635E–03
*H2AFY*	5.324E–01	1.549E–03
*CXCL14*	6.699E–01	7.222E–04
*C5orf20*	8.198E–01	2.064E–04
*PITX1*	8.438E–01	1.543E–04
*CDKL3*	8.716E–01	1.039E–04
*CDKN2AIPNL*	8.742E–01	9.965E–05
*TCF7*	9.262E–01	3.409E–05
*TIFAB*	9.610E–01	9.483E–06
*SKP1*	9.949E–01	1.604E–07

The nominal significant results were in bold.

## DISCUSSION

In post-GWAS era, the major challenge is to identify specific functional genetic variants that actually account for phenotypes and diseases [31]. With the analysis of ChIP-seq data of histone modifications, we previously screened out a potentially regulatory SNP rs17716310 associated with CRC risk. In current study, we verified rs17716310 as a functional variant of the chromosome 5q31.1. It showed that the minor C allele of rs17716310 conferred increased risk of CRC, improved transcriptional activity, and was associated with higher *SMAD5* expression in CRC tissue.

The findings led us to purpose that rs17716310 might influence CRC risk by altering the activity of an enhancer that control *SMAD5* expression. Lying within a region of the genome exhibiting H3k4me1 and H3k27ac, rs17716310 is highly suggested to locate in an active enhancer [30, 32, 33]. It is approximately 1Mb upstream of the gene *SMAD5*, which acts as an intracellular mediators that transduces signals of BMPs (bone morphogenetic proteins) belonging to TGF-β (transforming growth factor beta) superfamily [34–36]. It was reported that the Smad 1/5/8 signaling pathway could be activated by microRNA-1246, which was secreted from colorectal cancer cell-derived microvesicles, and thus be involved in the promotion of angiogenesis [37]. Recently, the inflammatory factor S100A8 was found to activate the Akt1-Smad5-Id3 axis, which promoted the proliferation, invasion and metastasis of colon cancer cells [38]. Moreover, the participation of TGF-β in colorectal tumorigenesis has been well recognized. The TGF-β signaling alterations mediated by variants of TGF-β receptors or SMADs contributed to colon cancer development and progression [39, 40]. On the other side, in a online database HaploReg [41], rs17716310 was indicated to change the binding motif of p300 that functions as a transcriptional coactivator and histone acetyltransferase regulating gene expression by remodeling chromatin [42]. Taken all together, the variant might improve the binding of some transcription factor(s) like p300, and stimulate the interaction between this active enhancer and the *SMAD5* promoter. It thereby activates the transcription of the effector *SMAD5*, and then strengthens the transduction of TGF-β signaling pathway that promotes the survival, invasion and metastasis of colorectal cancer cells [39].

As for the interaction with smoking found in multiplicative model, it might be due to the relations between cigarette smoke and the TGF-β pathway [43, 44] involving *SMAD5*. So, it is biologically plausible that rs17716310 could cooperate with smoking to increase CRC risk.

However, there are several limitations in this report. First of all, more functional experiments, such as electrophoretic mobility shift assays (EMSA) and real-time quantitative polymerase chain reactions (qPCR) were needed to verify our assumptions about the biological mechanism. Second, we did not study the association between the identified variant rs17716310 and metastasis or survival, because of the lack of clinical and prognostic information. Third, insufficient demographic and environmental data restricted us to adjust other influencing factors like diet, obesity, physical activity and drinking status in our statistical analyses.

In conclusion, integrating bioinformatics analysis, large-sample-size population association study and functional experiments, we highlighted a functional SNP rs17716310 for colorectal cancer risk mapping to chromosome 5q31.1. Systematic researches on more susceptibility loci are warranted to identify causal variants and elaborate the genetic etiology of CRC.

## MATERIALS AND METHODS

### Study participants

The present validation study included 1075 cases and 1999 controls, which were enrolled from 2011 to 2015 at the Tongji Hospital of Huazhong University of Science and Technology (HUST). All subjects were unrelated ethnic Han Chinese. The inclusion criteria for patients were histopathologically confirmed CRC without previous chemotherapy or radiotherapy. Cancer-free controls came from health check-up programs at the same hospital during the same time, which were matched to cases by gender and age (± 5 years). 1 ml peripheral blood was collected from each subject after a written informed consent was obtained, and demographic information including sex, age and smoking status were collected by interviewers. Herein, definitions of smoking status were the same as a previous study of our group [45]. And the population characteristics of discovery study were detailed in our previous report [30]. This study was conducted under the approval from the Institutional Review Board of Tongji Medical College of Huazhong University of Science and Technology.

### Genotyping

DNA was extracted from peripheral blood leukocytes with RelaxGene Blood System DP319-02 (Tiangen, Beijing, China). Candidate SNP was genotyped by the TaqMan SNP Genotyping Assay on an ABI PRISM 7900HT Fast Real-Time PCR platform (Applied Biosystems, Foster City, CA, USA). Quality control was preformed by including 5% duplicate samples in blinded fashion, with a concordance rate of 100%.

### Construction of reporter plasmids, transient transfections and luciferase assay

The 2001 bp DNA fragments, which were 1 kb upstream and downstream flanking A and C allele of rs17716310 (chr5: 134475759-134477759), were synthesized and cloned into Kpnl and Xhol restrictive sites of the pGL3-promoter vector (Promega) in both forward and reverse directions (Genewiz). The constructed plasmids were sequenced to verify the accuracy. HCT-116 and LoVo cells (1.25 × 10^4^ cells/well) were seeded into 96-well plates. Cells were co-transfected with 100 ng constructed vector (with the A allele or C allele) and 1ng pRL-SV40 Renilla luciferase plasmid (Promega) using Lipofectamine 3000 Reagent (Life Technologies) according to the manufacturer's instructions. After 24 hours, Firefly and Renilla luciferase activities were determined with the Dual-Luciferase Reporter Assay System (Promega). As the relative luciferase activity, the ratio of Firefly to Renilla luciferase activities was calculated for each sample. Three independent transfection experiments were carried out, and each experiment was conducted in triplicate. The data was presented as mean ± SD (standard deviation) and two-sided *P*-values were calculated using the Student's *t*-test.

### eQTL analyses

From the TCGA portal (http://cancergenome.nih.gov/) up to October 2014, we downloaded the data of gene expression, CpG methylation, somatic copy number and germline genotypes for COAD (colon adenocarcinoma) and READ (rectum adenocarcinoma). The correlations between the SNP and expression of genes within 1 Mb flanking regions was evaluated under a linear regression model with the effects of CpG methylation and somatic copy numbers being adjusted, according to the algorithms reported by Li *et al.* [46]. And the gene expression levels between CRC and peri-tumorous normal tissues were compared by independent-sample and paired-sample *t*-test.

### Statistical analysis

The differences in the distributions of demographic variables and genotype frequencies between cases and controls were estimated by χ^2^ test or *t*-test, where appropriate. Hardy-Weinberg equilibrium (HWE) of genotypes was evaluated in controls by a goodness-of-fit χ^2^ test. Odds ratios (ORs) and corresponding 95% confidence intervals (95% CIs) were calculated by unconditional multivariable logistic regression, with gender, age group and smoking status adjusted as categorical covariables. The potential gene-environment interaction was evaluated by a pair-wise analysis under multiplicative [47] and additive interaction models [48]. All statistical analyses, including the calculation of *P* values for multiplicative interaction under the multivariable logistic regression model, were performed with SPSS Software v20.0 (SPSS, Chicago, Illinois, USA), except the *P* values for additive interaction that were assessed by a bootstrapping test of goodness-of-fit using Stata v11.0 (Stata Corporation, College Station, TX). *P* values were two sided with the statistical significance criteria of *P* < 0.05 all through the study.

## SUPPLEMENTARY MATERIALS TABLE


