# Supplementary material for: Identification of a functional variant for colorectal cancer risk mapping to chromosome 5q31.1
Source: Oncotarget. 2016 May 11;7(23):35199–207. doi: 10.18632/oncotarget.9298 (PMC5085221; doi:10.18632/oncotarget.9298)
Supplement: Supplementary file 1 [file oncotarget-07-35199-s001.pdf]

Identification of a functional variant for colorectal cancer risk mapping to chromosome 5q31.1

Supplementary Materials

Supplementary Table S1: The association between rs17716310 and CRC risk by gender and median age in combined study

Subjects	Genotype	Control	Case	P^a	OR (95% CI)	P^b
Gender						
Male	AA	745	447	**0.022**	1.000	
	AC + CC	816	589		**1.223 (1.043–1.435)**	**0.013**
Female	AA	543	324	0.232	1.000	
	AC + CC	569	381		1.093 (0.903–1.324)	0.359
Median age						
Age ≤ 60.0	AA	619	381	0.567	1.000	
	AC + CC	737	477		1.051 (0.885–1.250)	0.569
Age > 60.0	AA	669	390	**0.002**	1.000	
	AC + CC	648	492		**1.302 (1.094–1.548)**	**0.003**

[a]*P* values were calculated by the Pearson Chi-Square test.
[b]Data were calculated by logistic regression model.
The nominal significant results were in bold.
